# Palm Fruit Bioactives augment expression of Tyrosine Hydroxylase in the Nile Grass Rat basal ganglia and alter the colonic microbiome

**DOI:** 10.1038/s41598-019-54461-y

**Published:** 2019-12-09

**Authors:** Robert P. Weinberg, Vera V. Koledova, Avinaash Subramaniam, Kirsten Schneider, Anastasia Artamonova, Ravigadevi Sambanthamurthi, K. C. Hayes, Anthony J. Sinskey, ChoKyun Rha

**Affiliations:** 1 Department of Biology, Massachusetts Institute of Technology, Cambridge, Massachusetts 02139 USA; 2 Biomaterials Science and Engineering Laboratory, Massachusetts Institute of Technology, Cambridge, Massachusetts 02139 USA; 3 Department of Biology, Brandeis University, Waltham, Massachusetts 02453 USA; 4 Advanced Biotechnology and Breeding Centre, Malaysian Palm Oil Board, 6, Persiaran Institusi, Bandar Baru Bangi, 43000 Kajang, Selangor Malaysia

**Keywords:** Enzymes, Neuronal physiology, Neurology, Medical research

## Abstract

Tyrosine hydroxylase (TH) catalyzes the hydroxylation of L-tyrosine to L-DOPA. This is the rate-limiting step in the biosynthesis of the catecholamines – dopamine (DA), norepinephrine (NE), and epinephrine (EP). Catecholamines (CA) play a key role as neurotransmitters and hormones. Aberrant levels of CA are associated with multiple medical conditions, including Parkinson’s disease. Palm Fruit Bioactives (PFB) significantly increased the levels of tyrosine hydroxylase in the brain of the Nile Grass rat (NGR), a novel and potentially significant finding, unique to PFB among known botanical sources. Increases were most pronounced in the basal ganglia, including the caudate-putamen, striatum and substantia nigra. The NGR represents an animal model of diet-induced Type 2 Diabetes Mellitus (T2DM), exhibiting hyperglycemia, hyperinsulinemia, and insulin resistance associated with hyperphagia and accelerated postweaning weight gain induced by a high-carbohydrate diet (hiCHO). The PFB-induced increase of TH in the basal ganglia of the NGR was documented by immuno-histochemical staining (IHC). This increase in TH occurred equally in both diabetes-*susceptible* and diabetes-*resistant* NGR fed a hiCHO. PFB also stimulated growth of the colon microbiota evidenced by an increase in cecal weight and altered microbiome.  The metabolites of colon microbiota, e.g. short-chain fatty acids, may influence the brain and behavior significantly.

## Introduction

### Tyrosine Hydroxylase - A Key Monooxygenase in Catecholamine Biosynthesis

**Figure 1:**
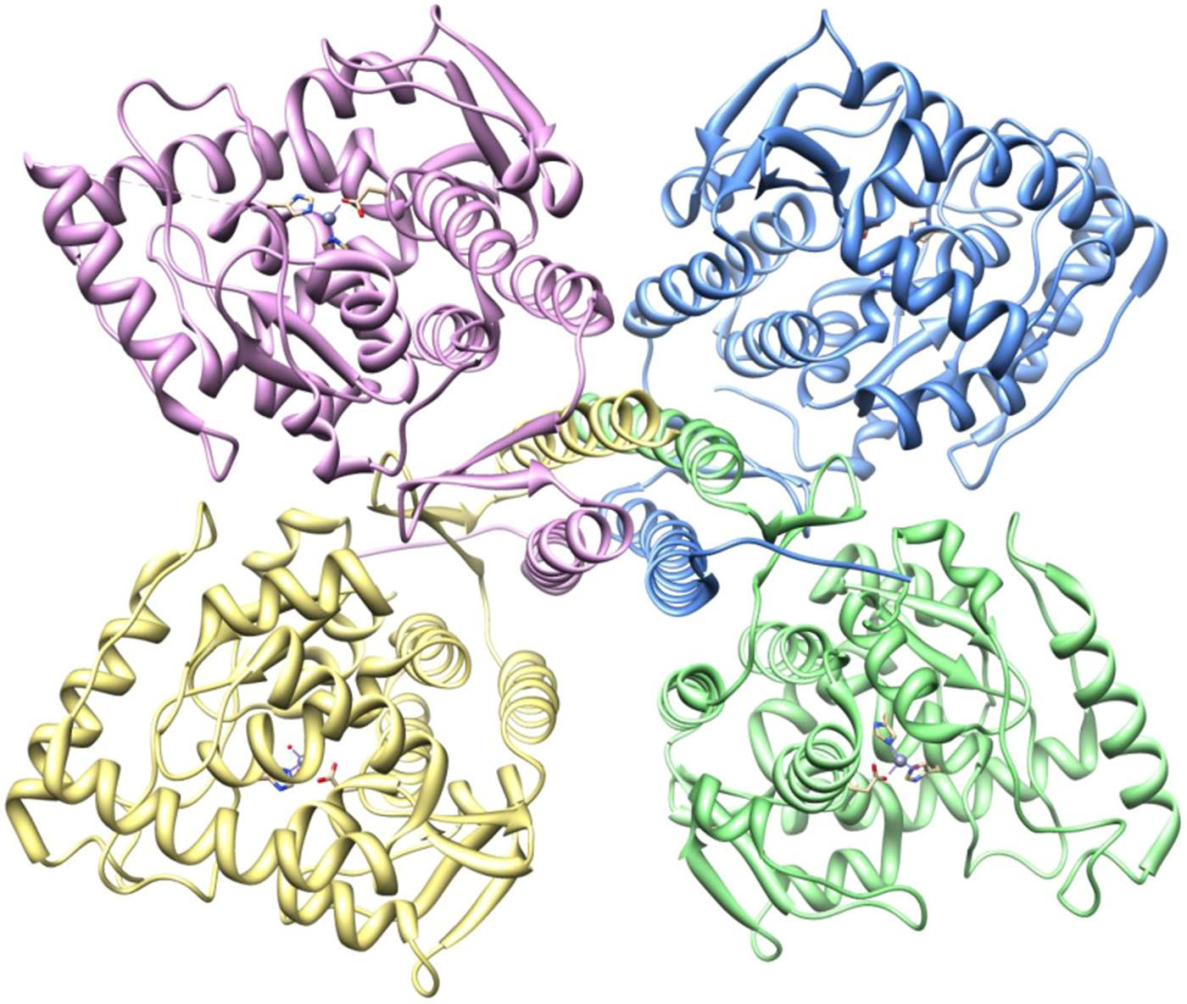
Tertiary structure of Tyrosine Hydroxylase using UCSF Chimera 1.6.1/PDB ID 2xsn. [Tyrosine hydroxylase showing all four subunits.png] *by Gla086 licensed under CC BY-SA 3.0* Link: https://search.creativecommons.org/photos/d3c70e04-ce8f-4974-a747-00933c48925d.

**Figure 2:**
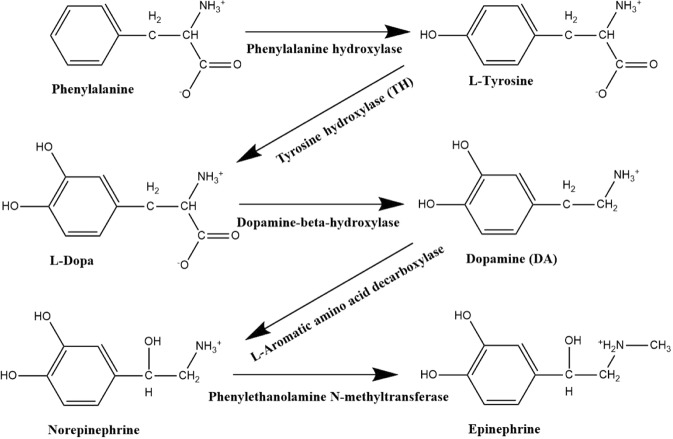
Mammalian catecholamine biosynthetic pathway from Phenylalanine. Phenylalanine hydroxylase converts phenylalanine to tyrosine. Tyrosine hydroxylase hydroxylates tyrosine to L-DOPA. L-DOPA is converted to dopamine by aromatic amino acid decarboxylase. Dopamine-β-hydroxylase hydroxylates dopamine to norepinephrine, which is then methylated to epinephrine by phenylethanolamine N-methyltransferase.

Tyrosine hydroxylase (TH) [EC# 1.14.16.2] is a tetrahydrobiopterin-requiring monooxygenase (Fig. 1) which catalyzes the hydroxylation of L-tyrosine to L-DOPA (L-dihydroxyphenylalanine) which represents the rate-limiting step in the biosynthesis of the catecholamines (CA) including dopamine (DA), norepinephrine (NE) and epinephrine (EP)^1–5^. The catecholamines DA, EP and NE are key neurotransmitters and hormones in the mammalian central and peripheral nervous systems. TH activity is tightly regulated at both the transcriptional and post-transcriptional levels^6–13^. The biosynthetic pathway for CA is depicted in Fig. 2.

Physiologic functions of the catecholamines include essential roles in cognition^14^, memory^15^, attention^16^ and emotion^17,18^. The neuronal cell bodies of the CNS catecholamine-producing neurons are primarily localized to the brain stem and have been extensively mapped by immunohistochemical methods^19^. Multiple medical conditions result from aberrant expression or activity of CA including bipolar disorder^20^, hypertension^21^, drug addiction^22^, schizophrenia^23^, muscle dystonias^24^ and Parkinson’s disease^25^.

## Background

### The Nile grass rat metabolically mirrors diabetes mellitus in humans

The Nile Grass rat (NGR), *Arvicanthis niloticus*, has demonstrated itself to be a unique animal model for diet-induced Type 2 Diabetes Mellitus (T2DM) because the NGR exhibits many of the pathologic features of T2DM^26–38^. When fed a fiber-free carbohydrate-rich diet, or even a standard commercial rodent chow, its carbohydrate and energy metabolism becomes deranged, in a manner similar to Metabolic Syndrome (MetS) and T2DM in humans.

The weanling NGR that develops MetS, experiences hyperphagia and accelerated weight gain which evolves into T2DM within 8–10 weeks while fed a high carbohydrate diet (as opposed to its natural high fiber diet in its native environment). The MetS-T2DM syndrome in the NGR is also characterized by hyperinsulinemia, hyperglycemia, insulin resistance, hyperlipidemia and hepatic pathology resembling non-alcoholic fatty liver disease (NAFLD) in humans as well as developing renal pathology culminating in renal failure^26–38^. Few other animal models mimic the T2DM observed in humans with such similar pathologic features of T2DM, making the NGR model of MetS/T2DM a unique animal model.

### Association of Alzheimer’s disease with diabetes mellitus

Preliminary observations made by the Hayes Lab identified locomotor deficits in the NGR afflicted with advanced T2DM including ataxia, limb weakness, incoordination and gait disturbance, all of which suggested some degree of CNS-locomotor involvement. Furthermore, several decades of epidemiologic research in humans suggested an association between Alzheimer’s disease and T2DM ^39–49^. Thus the NGR was chosen for investigation of its brain, based on the hypothesis that the NGR advanced T2DM state may be linked to brain neuropathologic changes similar to those found in the brains of humans with T2DM associated with Alzheimer’s disease. If such an association were true, the NGR may offer a unique opportunity to investigate the effects of PFB on the pathogenesis of such neuropathology because multiple prior studies have shown that the NGR T2DM/MetS   and its sequellae are reduced by PFB supplementation^50–52^.

Other studies by the Rha Lab have shown that PFB can inhibit the aggregation and fibrillization of beta-amyloid peptide *in vitro* ^53^ and that PFB significantly modulates the cytokine secretome of IL-1β-activated human astrocytes *in vitro*, with significant reduction of TNF-α, RANTES and IP-10^54^. These actions of PFB on beta-amyloid and astrocyte cytokines *in vitro* provide a potential model for investigating novel therapeutics aimed at reducing neuroinflammation in the neurodegenerative diseases. Furthermore, recent studies in the Sinskey Lab have shown that PFB reduces the cytotoxicity of aggregated α-synuclein peptide overexpressed in a transgenic yeast rescue assay [Angela Botes and Christian Ruckert, personal communication].

### Palm fruit bioactives are an aqueous extract from the oil palm fruit

**Figure 3:**
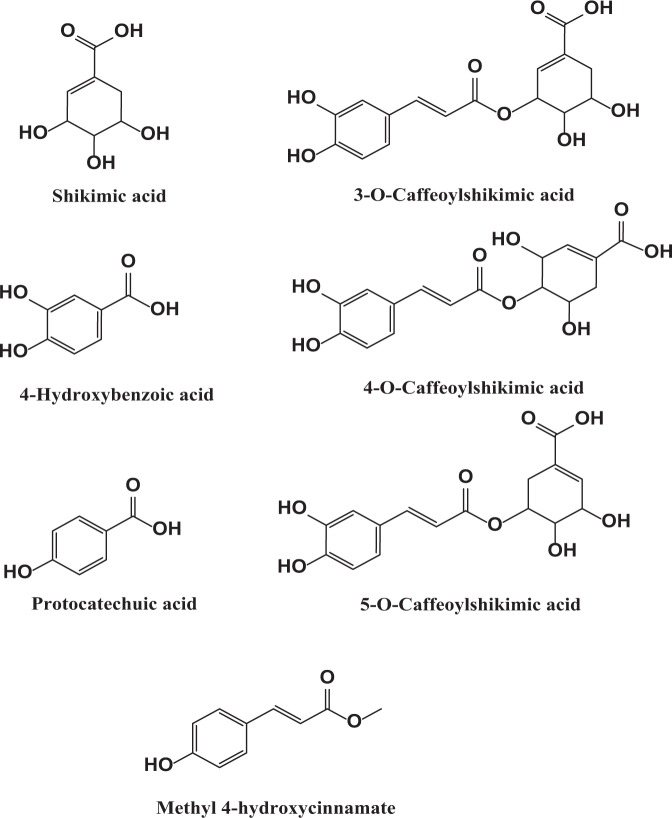
Some signature bioactive organic compounds present in PFB. PFB is a complex heterogeneous phytochemical mixture prepared from an aqueous extract from the fruit of the oil palm tree.

PFB is an aqueous extract from the fruit of the oil palm tree, *Elaeis guineensis*^55–60^. Several signature bioactive organic compounds have been identified and are depicted in Fig. 3. The five signature polyphenol compounds include protocatechuic acid, 4-hydroxybenzoic acid, and the three isomers of 3-caffeoylshikimic acid, 4-caffeoylshikimic acid, and 5-caffeoylshikimic acid, along with shikimic acid and methyl-4-hydroxycinnamate. HPLC and LS/MS/MS have shown that PFB also includes smaller amounts of gallic acid, vanillic acid, caffeic acid, syringic acid, *p*-coumaric acid and ferulic acid. Depending on batch-specific processing of the crushed-steamed fruit, PFB has approximately 5–10% dissolved solids in aqueous solution, of which about 2% are antioxidant polyphenols. These polyphenol compounds have significant antioxidant properties^61^ as determined by the 2,2-diphenyl-1-picrylhydrazyl (DPPH)^62^ assay as well as by the Gallic Acid Equivalent (GAE)^63^ assay.

Multiple published studies have been performed with PFB (a.k.a. “Oil Palm Phenolics” and “Palm Fruit Juice”) which have shown the diverse biologic effects associated with PFB (Table 1).

**Table 1:** Biologic Effects associated with Palm Fruit Bioactives (a.k.a. Oil Palm Phenolics).

Biologic Effect	Model System	Discovery
Reduction in hyperglycemia, increased insulin sensitivity, reduced MetS & T2DM pathology	Nile Grass rat	Bolsinger *et al*.^50^
Reduction in arterial blood pressure via increased endothelial production of nitric oxide	Rabbit	Sambanthamurthi *et al*.^65^
Reduction of reactive oxygen species via direct neutralization and increased oxido-reductases	*In vitro*	Sambanthamurthi *et al*.^65^
Inhibition of cancer cell proliferation and growth; inhibition of proliferation and growth of xenotransplanted tumors	Mouse	Sambanthamurthi *et al*.^51^ Sekaran^77^ Ji *et al*.^78^
Reduction in retroviral replication via inhibitory action against HIV protease and reverse transcriptase	*In vitro*	Sambanthamurthi *et al*.^79^
Modulation of IL-1β-activated astrocyte cytokine secretome with reduction of TNF-α, RANTES, IP-10	*In vitro*	Weinberg *et al*.^54^
Reduction in beta-amyloid (1–42) peptide aggregation and reduced fibrillization	*In vitro*	Weinberg *et al*.^53^
Reduction in mitochondrial genome mutation rate following exposure to genotoxins	*In vitro*	Osborne *et al*.^80^
Inhibition of angiogenesis and inflammation	Mouse	Zandi *et al*.^52,81^
Alteration of GI motility & physiology	Rat	Patten *et al*.^82^
Reduction of the pro-inflammatory transcription factor NF-kB	*In vitro*	Ji *et al*.^78^
Alteration of colonic microbiota with differential bacterial species & populations	Rat	Conlon *et al*., submitted
Radio-protection and increased cellular resistance to high-energy ionizing radiation	*In vitro*	Koledova *et al*.^83^
Modulation of the murine transcriptome	Mouse	Leow *et al*.^64^
Reduction in LDL oxidation	*In vitro*	Sambanthamurthi *et al*.^65^
Reduction of ischemic cardiac ventricular arrhythmias induced by ligature of coronary arteries	Rat	Sambanthamurthi *et al*.^51^
Reduction of arterial atherosclerosis	Rabbit	Idris *et al*.^84^
Alteration of hormetic stress response genes with increase in fruit fly longevity	Fruit Fly	Leow *et al*.^85^
Increase in neuroprotection and enhancement of cognitive processes	Mouse	Leow *et al*.^64^
Reduction of plasma lipids and reduced obesity via altered metabolic energy profile	Mouse	Sambanthamurthi *et al*.^65^

 Research by the Malaysian Palm Oil Board has also demonstrated potent antioxidant activities of PFB on both molecular and genetic levels including the direct free radical inactivation *in vitro* as well as the upregulation of specific phase II hepatic detoxifying enzymes, upregulation of heme-containing oxido-reductases which reduce the levels of reactive oxygen species (ROS) *in vitro*, as well as increases in the level of intracellular glutathione due to increased activity of glutathione synthetase and glutathione reductase^57,59,61^. Based on these studies, the antioxidant potency of PFB is superior to   that of curcumin, ascorbate and beta-carotene.

## Results

### Biochemical and physiological changes in Nile grass rats fed a high CHO diet

Thirty-nine male NGR were weaned at 3 weeks and fed a semi-purified high-carbohydrate diet (hiCHO) [60:20:20, carbohydrate:fat:protein, as percent calories] with or without 10% PFB (w/w) supplemented as a spray-dried powder incorporated in the diet to give a final concentration of 4.7 mg GAE/g (GAE = gallic acid equivalents) diet for 8 weeks duration. This hiCHO diet has been previously shown to induce MetS and T2DM in this NGR model^26–28^. Table 2 lists some of the physiological parameters affected by diet and shows that this 60:20:20 diet induced diabetes in 57% (12/21) of the 21 rats fed the hiCarb diet without PFB. By contrast, only 33% (6/18) of the rats supplemented with PFB developed diabetes, which represents a 45% reduction in T2DM.

**Table 2:** Physiologic parameters for 39 male NGR [separated at 3 wks old] fed a semi-purified high carbohydrate diet (60:20:20) with or without 10% PFB for 8 weeks, then subdivided into *resistant* or *susceptible* to diabetes based on a random blood glucose (RBG) ≥ 75 mg glucose/100 ml plasma.

Ingredient	Diet			
	No PFB		10% PFB	
CHO:Fat:Prot %E	**60:20:20**		**60:20:20 + 10%PFJ**	
kcal/g	**4.2**		**4.2**	
mg GAE/kcal	**0.0**		**1.14**	
mg GAE/g diet	**0.0**		**4.8**	
	**resistant**	**susceptible**	**resistant**	**susceptible**
	n=21		n=18	
	**<75 mg**	**>75 mg**	**<75 mg**	**>75 mg**
**RBG >75 mg/dl** **(% incidence T2DM) n=**	9	12 (66%)	12	6 (33%)
**Random Body weight (g)**				
Initial (3wk of age)	28 ± 3	28 ± 4	28 ± 4	30 ± 6
After 4 wks	71 ± 8	78 ± 6^a^	70 ± 7^a^	74 ± 9
After 8 wks	92 ± 5	97 ± 7^a^	90 ± 9^a,b^	99 ± 8^b^
**Random blood glucose (mg/dl)**				
After 4 wks	68 ± 6^a^	125 ± 88^a,b^	76 ± 17^b^	89 ± 34
After 8 wks	63 ± 5^a^	268 ± 209^a,b^	63 ± 7^b^	163 ± 162
**OGTT (BG mg/dl)**				
After 4 wks				
Fasting blood glucose, 0 min	55 ± 25	54 ± 18	57 ± 9	46 ± 6
30 min	230 ± 48	261 ± 43^a^	198 ± 59^a^	221 ± 79
After 8 wks				
Fasting blood glucose, 0 min	47 ± 8	59 ± 27	57 ± 11	55 ± 12
30 min	182 ± 27^a,b,c^	372 ± 101^a,d,e^	263 ± 63^b,d^	306 ± 94^c,e^
60 min	97 ± 32^a,b^	269 ± 141^a,c^	165 ± 48^c^	202 ± 112^b^
**Organ weight** (%BW)				
Liver	3.16 ± 0.26^a^	3.80 ± 0.55^a,bc^	3.46 ± 0.20^b^	3.43 ± 0.22^c^
Kidne	0.66 ± 0.05^a^	0.73 ± 0.07^a^	0.70 ± 0.04	0.72 ± 0.11
Cecum	1.16 ± 0.12^a,b^	1.29 ± 0.28^c,d^	1.64 ± 0.40^a,c^	1.58 ± 0.15^b,d^
Adipose				
Epididymal	3.31 ± 0.48^a,b^	2.86 ± 0.38^a^	2.81 ± 0.50^b^	3.07 ± 0.40
Perirenal	1.54 ± 0.19	1.69 ± 0.52	1.56 ± 0.40	1.64 ± 0.38
Brown fat	1.73 ± 0.36	2.00 ± 0.34	1.88 ± 0.41	2.05 ± 0.31
Total fat	6.59 ± 0.64	6.56 ± 1.04	6.26 ± 1.05	6.76 ± 0.49
Carcass	75 ± 1	76 ± 7	74 ± 1	74 ± 1
**Body length (cm)**	12.9 ± 0.5	13.4 ± 0.6^a^	12.9 ± 0.6^a^	13.3 ± 0.5

Values are Mean ± SD; ^a^Significant (P < 0.05) interaction term for diet × diabetes class (RBG <75 mg/dl>) revealed by two-way ANOVA; ^b^Significant (P < 0.05) effect of diet (GLoad) by two-way ANOVA; ^c^Significant (P < 0.05) increase due to diabetes (RBG > 75 mg/dl) by two-way ANOVA; ^d^Significant (P < 0.05) decrease due to diabetes (RBG > 75 mg/dl) by two-way ANOVA; All serum analyte statistical analyses  were carried out by two-way ANOVA . ND - Not detectable, below level of detection.

T2DM was defined by an elevated random blood glucose (RBG) and fasting blood glucose (FBG) in the plasma, as well as by an abnormal oral glucose tolerance test (OGTT) at 4 weeks and 8 weeks post-weaning. The NGR is characterized by a *genetic permissiveness* toward the development of T2DM. While fed a highCHO diet, the NGR can be divided into 2 groups: diabetes-*susceptible* and diabetes-*resistant*.

Accordingly, none of these abnormal glucose values which were noted in the *susceptible* rats were apparent in the diabetes-*resistant* group. An abnormal plasma glucose profile in the *susceptible* rats was further supported by gross anatomical changes observed at necropsy. For example, the liver and kidney gross weights were greater in diabetes-*susceptible* rats, due to fat infiltration and renal dysfunction, as compared to diabetes-*resistant* animals. Measures of daily food intake positively linked total calories consumed to body weight gain, RBG, FBG and the OGTT at 4 weeks and 8 weeks (Table 2).

These metabolic parameters were also related to the organ pathology observed at necropsy after 8 weeks. Rats from both the diabetes-*susceptible* and diabetes*-resistant* subgroups were randomly assigned to a semipurified diet either supplemented with 10% PFB or without supplementation, upon initiation of the experiment at the time of weaning. The most notable response, summarized earlier, was that fewer rats supplemented with PFB developed diabetes (33% vs 57%). Six rats that developed diabetes while supplemented with PFB revealed less severe symptoms and fewer abnormal parameters (Table 2).

### Effects of PFB on the NGR rat brain in diabetes-susceptible vs diabetes-resistant rats

Early observations by the Hayes Lab of NGR with advanced diabetes fed hiCHO revealed locomotor dysfunction with impaired balance and gait, ataxia, tremors, increased aggressiveness and occasional seizures when alarmed. However, the NGR which were supplemented with PFB were spared from these neurologic impairments in addition to the reduction in diabetic metabolic impairment including better overall glucose control, less hyperglycemia, better insulin levels and less insulin resistance as previously reported^26–28^. For the past 2 decades, the diabetic literature has suggested a link between T2DM and neurodegenerative diseases (NDG) such as Alzheimer’s disease^39–49^. Based on this association of T2DM and Alzheimer’s disease in humans along with the observed locomotor neurologic impairment of the NGR rats with advanced diabetes, we decided to investigate the brains of the NGR fed the hiCHO diet, looking for histological evidence of neurodegenerative disease similar to those seen in humans, involving aggregates of aberrant proteins such as beta-amyloid, alpha-synuclein, hyperphosphorylated tau or aberrant neuronal enzymes.

### Anatomic and Histologic changes seen in the brains of NGR rats by immunohistochemistry

Antibodies were chosen from a commercially-available Immunohistochemical Neurologic Panel which included antibodies against beta-amyloid peptide, phosphorylated tau proteins, alpha-synuclein, as well as tyrosine hydroxylase and other neuronal enzymes represented in the panel. Table 3 lists the semi-quantitative results of this IHC staining for β-amyloid, phosphorylated tau protein, α-synuclein and tyrosine hydroxylase in the brains of adult NGR rats with or without PFB supplementation. IHC staining was also performed against other neuropeptide and enzyme targets with negative results.

**Table 3:** Semi-quantitative comparisons of CNS proteins by IHC staining.

Protein	DM-resistant, without PFB	DM-resistant, with PFB	DM-susceptible, without PFB	DM-susceptible, with PFB
Tau	2+	2+	2+	2+
β- amyloid	1+	+/−	1+	+/−
α-synuclein	3+	3+	3+	3+
Tyrosine hydroxylase	1+		1+	7+

Minimal differences detected for levels of beta-amyloid, tau protein, or alpha-synuclein between diabetes-susceptible and diabetes-resistant rats, irrespective of whether they were supplemented with PFB or not. However, Tyrosine Hydroxylase was significantly more positive in rats supplemented with PFB compared to those not supplemented. The presence or absence of diabetes [high blood glucose] did not appear to affect tyrosine hydroxylase expression.

Table 3 shows minimal differences detected by IHC staining for levels of beta-amyloid, phosphorylated tau protein, or alpha-synuclein between diabetes-susceptible and diabetes-resistant rats, irrespective of whether they were supplemented with PFB or not. However, IHC staining for Tyrosine Hydroxylase was significantly more positive in rats supplemented with PFB compared to those not supplemented. The presence or absence of diabetes [high blood glucose] did not appear to affect tyrosine hydroxylase expression.

These results disprove our original hypothesis that diabetes is associated with neurodegenerative  changes in the brain, looking at beta-amyloid, phosphorylated tau and alpha-synuclein, and leave us with the new hypothesis that PFB augments expression of TH in the brain regardless of the diabetic state.

Based on the positive IHC staining for TH in the NGR rat brains (Table 3), further histologic studies were performed on the brains of 21 male NGR rats (Table 4). Animals were divided based on diabetic metabolic status as either *susceptible* or *resistant* subgroups, with diabetes determined by an RBG greater than 75 mg/dl and FBG greater than 60 mg/dl.

**Table 4:** TH staining of the striatum in the brain by IHC was performed on these 21 NGR rats which were fed semipurified hiCHO diets (60:20:20) with or without 10% PFB for 8 weeks, which were subdivided into diabetes-susceptible or diabetes-resistant rats based on an RBG ≥ 75 mg/dl.

PFB status	Diabetes status	n	Mean optical density*
No PFB	Diabetes resistant	5	0.14 ± 0.04^a^
	Diabetes susceptible	5	0.16 ± 0.03^b^
	Severe diabetes	3	0.15 ± 0.06
+PFB 10%	Diabetes resistant	4	0.42 ± 0.09^a^
	Diabetes susceptible	4	0.38 ± 0.07^b^

^a,b^Significant difference (p < 0.05),  by two-way ANOVA.

*Mean optical density is proportional to the concentration of the stain. Optical density = mean ± SEM.

Image mean density collected by Nikon software from area ~5000 nm^2^ from subsection of the positively stained TH area (striatum).

Table 4 reveals the mean intensity of IHC staining for TH in the striatum by light microscopy for the 21 NGR rat brains, where the mean optical density represents the densitometry of the image analyzed using the Nikon NIS-elements BR software which determines staining intensity by measuring pixel density. For the NGR not supplemented with PFB, the mean optical density ranges from 0.14 for the diabetes-resistant, through 0.15 for severe diabetes, to 0.16 for the diabetes-susceptible. In contrast, for the NGR supplemented with PFB, the mean optical density ranges from 0.38 for the diabetes-susceptible to 0.42 for the diabetes-resistant.

**Figure 4:**
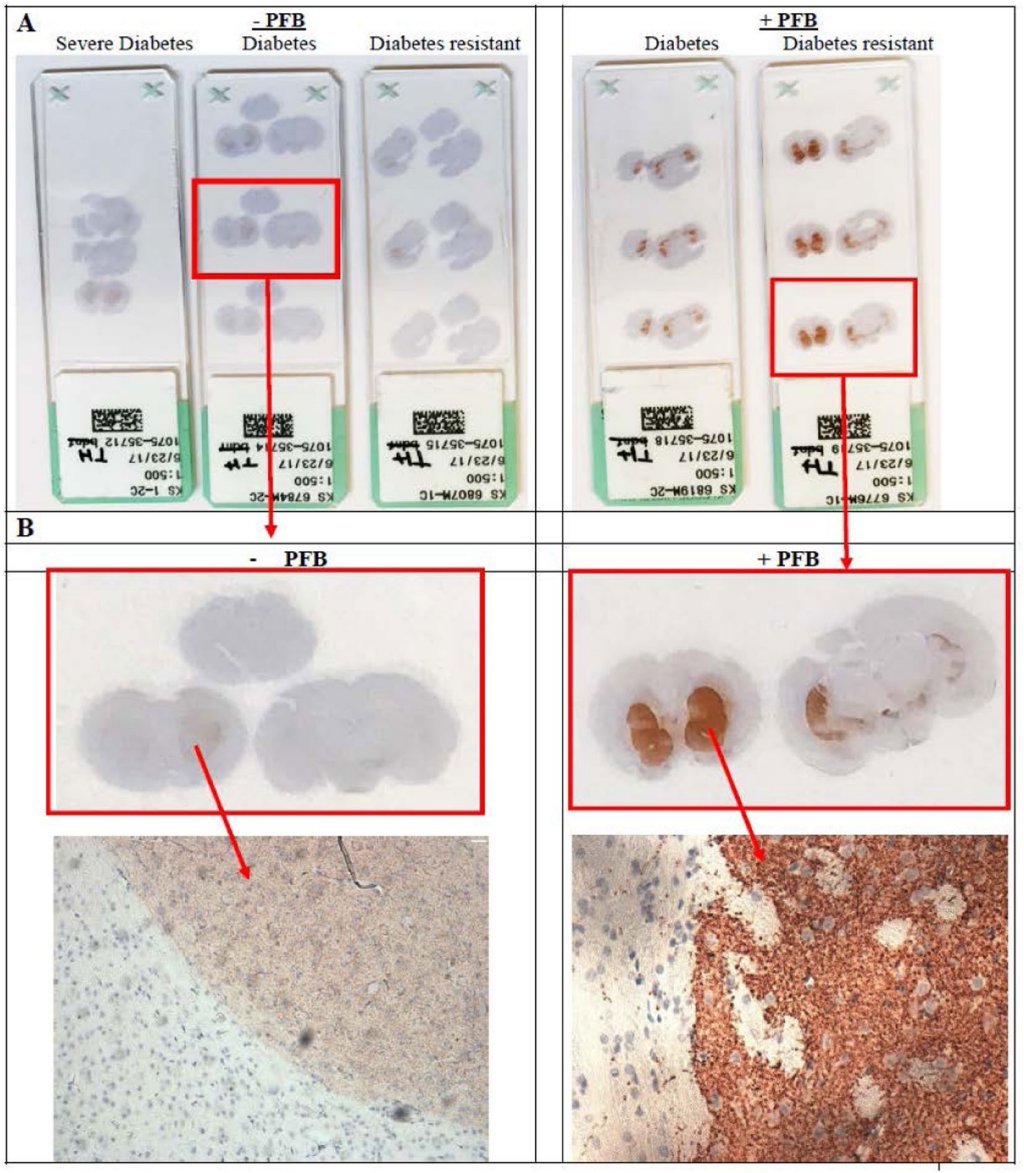
Coronal brain sections stained for TH by IHC. Rats with PFB (on right), show strong positive staining of the brain striatum, compared with rats not supplemented with PFB (on left). TH = brown color; nuclei counterstained with hematoxylin = blue color.

**Figure 5:**
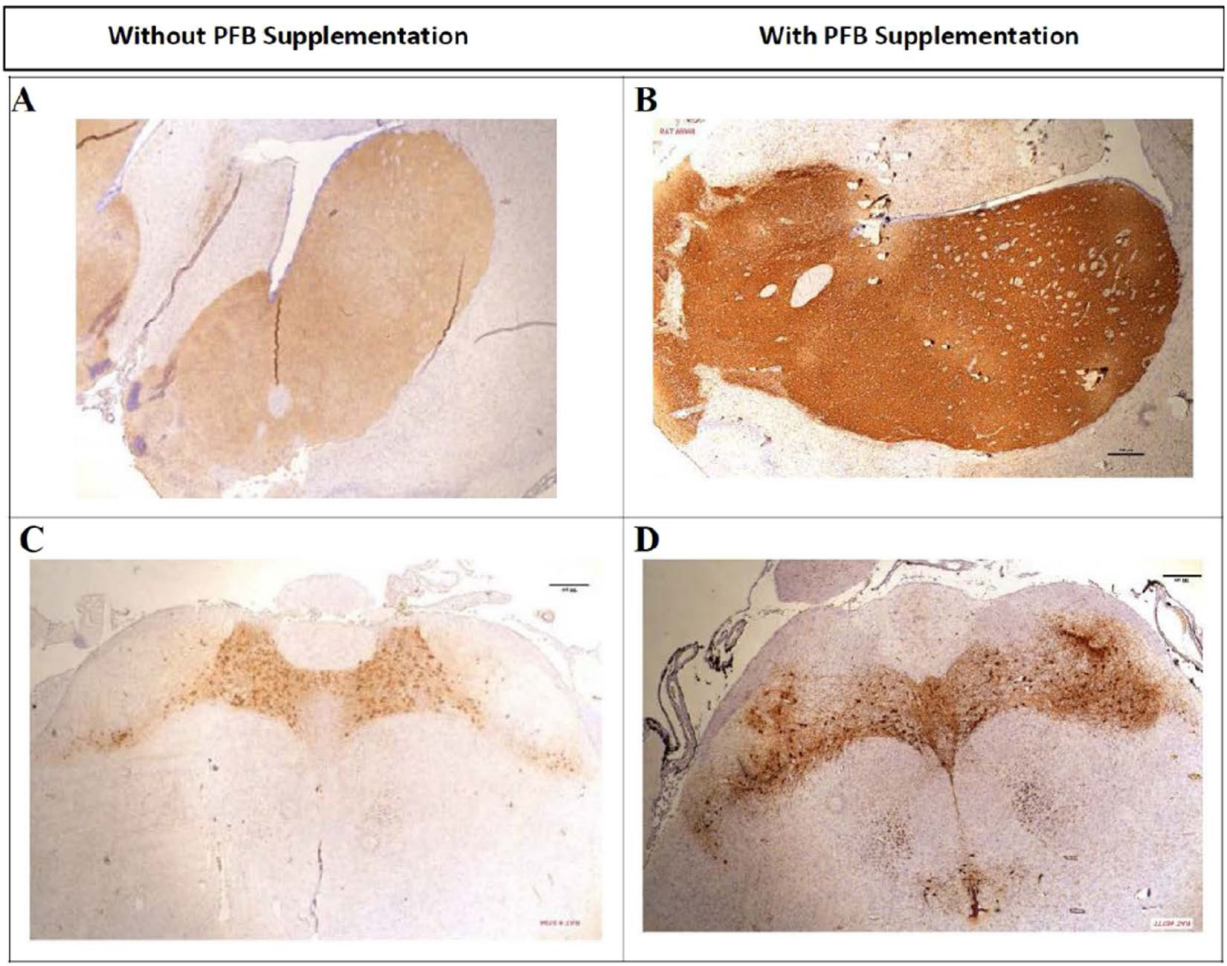
Coronal brain sections through Striatum and Substantia nigra compacta stained for TH by IHC. Panels A and B show Striatum; C and D show Substantia nigra compacta. (**A**,**C**) are sections from NGR fed hiCHO without PFB; B and D are sections from NGR fed hiCHO with PFB. There is strong positive staining for TH in (**B,D**), with only weak staining for TH in (**A,C**).

**Figure 6:**
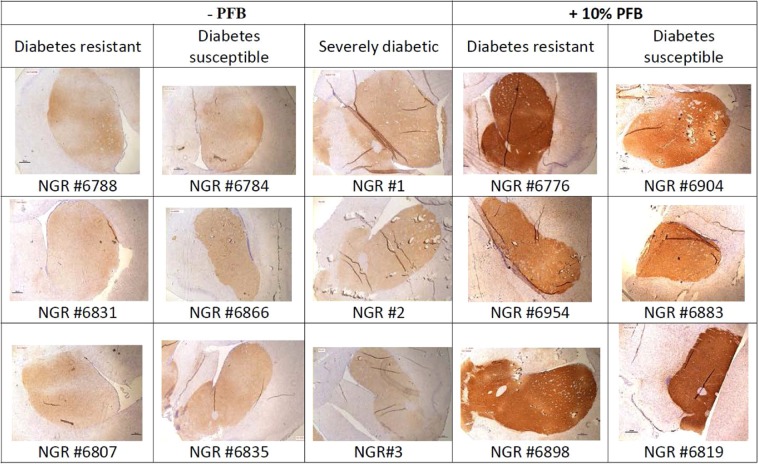
Additional coronal brain sections from 15 NGR stained for TH by IHC. The left 3 columns come from NGR not supplemented with PFB, and the right 2 columns are from NGR supplemented with PFB in their diet. The rats consumed on average 7.7 g of diet per rat per day, which was equivalent to 36.2 mg GAE. Based on average body weight at 8 weeks of 93 g, this was equivalent to 389 mg GAE per kg rat per day.

IHC staining was then performed on serial coronal sections of the NGR rat brains using a primary mouse anti-tyrosine hydroxylase antibody. IHC staining with anti-tyrosine hydroxylase antibody showed strong positive staining of the brain striatum, caudate-putamen and substantia nigra in the NGR rats whose diet was supplemented with PFB compared with those rats whose diet was not supplemented with PFB regardless of diabetic versus non-diabetic state (Figs. 4, 5 and 6).

### Distribution of tyrosine hydroxylase in serial coronal sections of the rat brain

On the right side of Fig. 4, we see that NGRs which were supplemented with PFB show strong positive staining of the brain striatum with anti-tyrosine hydroxylase antibody, compared with NGRs which were not supplemented with PFB, seen on the left side. These sections corroborate the novel finding that PFB induces increased expression of TH in the NGR brain, in this case involving the striatum.

Figure 5 shows coronal brain sections of the NGR through the striatum and substantia nigra which were stained for TH by IHC. The right side of Fig. 5(B,D) shows brain sections from NGRs which were supplemented with PFB, and we see strong staining for TH in the striatum (panel B) and the substantia nigra (panel D). The left side of Fig. 5(A,C) shows brain slices from those NGRs which were not supplemented with PFB, and there is only faint basal staining for TH in the striatum (panel A) or in the substantia nigra (panel C).

Figure 6 shows additional coronal brain sections of the striatum and caudate-putamen stained by IHC for TH in an additional 15 NGR rats. The left 3 columns were taken from rats without PFB supplementation and show faint basal staining for TH. The right 2 columns are from NGR supplemented with PFB and show strong dark intense staining for TH. Both groups consumed on average 7.7 g of diet per day. For the +10% PFB NGR, the 7.7 g of diet contained the equivalent of  36.2 mg GAE (antioxidant equivalents). Based on an average body weight of 93 g at 8 weeks, this dose was equivalent to 389 mg GAE per kilogram per day. Interestingly, the blood glucose levels (RBG, FBG) and diabetic state of resistance versus susceptibility had no effect on the expression of tyrosine hydroxylase in these brains. For increased TH expression, the only determinative factor was whether the NGR rats were supplemented with PFB or not.

### Quantitative analysis of IHC staining intensity by NIS-BR densitometry

Images were quantitatively analyzed by light microscopy and photo-densitometry using the Nikon NIS-elements BR software which measures the anti-TH staining intensity. All NGR brain sections revealed that supplementation of the diet with 10% PFB significantly increased the level of expression of tyrosine hydroxylase in the basal ganglia of the NGR brains. The staining for TH was most pronounced in the striatum, the caudate-putamen and the substantia nigra. No difference was seen between the diabetic and the non-diabetic rats. Our original hypothesis that the diabetic rats would have greater neuropathology than the non-diabetic rats was incorrect.

Imaging the IHC-stained striatum for TH revealed no significant difference between the diabetes-resistant and diabetes-susceptible or severe diabetes NGR rats (p > 0.05), with imaging intensities of: 0.14 ± 0.04, 0.16 ± 0.03 and 0.15 ± 0.06, respectively (Table 4). Also, no difference was observed between the imaging intensities of diabetes *susceptible* or *resistant* subgroups which did receive PFB supplementation with intensities of 0.42 ± 0.09 and 0.38 ± 0.07 units, respectively (p > 0.05) (Table 4).

**Figure 7:**
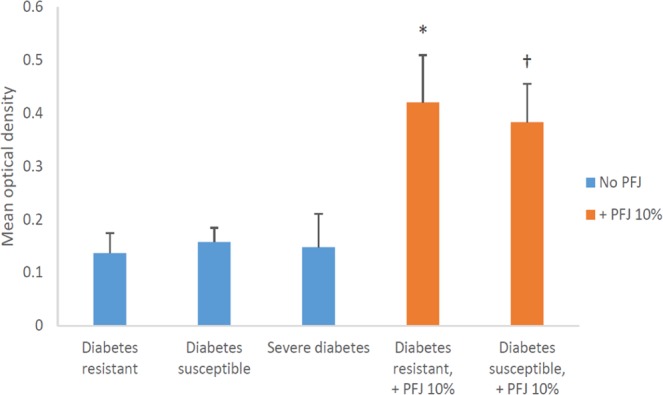
Quantification of TH staining in the Striatum by Nikon NIS-elements imaging software based on the density of pixels in image. (PFJ = PFB).

**Figure 8:**
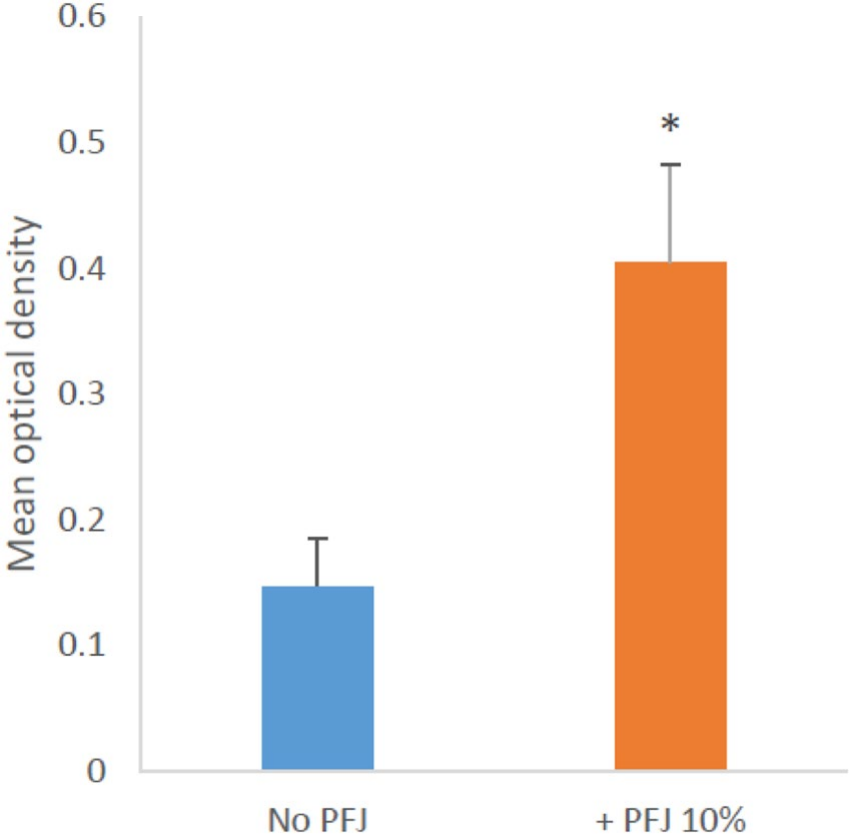
Quantification of staining intensity for tyrosine hydroxylase in the striatum by Nikon NIS-elements software showed a substantial increase in the level of TH in the striatum of the NGR receiving PFB supplementation.

However, a significant difference was found for TH between the NGR subgroup that received PFB supplementation versus those that did not receive PFB supplementation (p < 0.05) independent of diabetic metabolic state (Fig. 7 and Table 4). Specifically, PFB supplementation increased the expression of TH in the striatum of *diabetes-resistant* NGR rats 3-fold (p < 0.005) and 2.4-fold (p < 0.003) in *diabetes-susceptible* rats via Student’s t-test. Figure 8 and Table 5 compare the intensity of TH staining in the NGR brain combining all NGR rats that received PFB supplementation [0.40 ± 0.08 units] vs. those that did not [0.15 ± 0.04 units], (p < 0.05).

**Table 5:** Mean TH throughout the basal ganglia by IHC staining on 20 male Nile Rats (3 weeks old) fed semipurified hiCHO diets (60:20:20) with or without 10% PFB for 8 weeks or fed CHOW diets without PFB until the development of severe diabetes.

PFB supplementation	n	Mean optical density*
No PFB	13	0.15 ± 0.04*
+PFB 10%	7	0.40 ± 0.08*

*Mean optical density is proportional to pixel density of the stain. Mean optical density = mean ± SEM.Image mean density collected by Nikon software from area ~5000 nm^2^ from subsection of the positively stained TH area (striatum).Statistical analysis by Student t-Test.

In Table 5, we see that the seven NGR rats which received PFB have a mean optical density of 0.40 which is between 2 and 3 times as much tyrosine hydroxylase staining in the basal ganglia compared with the thirteen NGR rats which did not receive PFB with a mean optical density of 0.15.

Figure 8 shows us graphically that the NGR rats receiving PFB have substantially greater tyrosine hydroxylase staining than the NGR rats which did not receive PFB.

### Effects of PFB on NGR gastrointestinal physiology and the microbiome

The nutritional data and induction of T2DM observed were consistent with the NGR model of T2DM reviewed elsewhere^26–28^. In summary, the rats under study revealed differences in *genetic permissiveness* to diabetes when consuming the same hiCHO diet (60:20:20; % energy intake), including more rapid weight gain in the genetically-permissive NGR associated with the major elevation in random blood glucose (RBG) and the hyperglycemic oral glucose tolerance test (OGTT). It was previously shown that this reflects greater caloric intake by the *susceptible* rats. On the other hand, PFB had no detectable impact on weight gain, but it did reduce the *incidence* (−40% in RBG) and *severity* (−20% based on OGTT) of the hyperglycemia (surrogate for diabetes). As shown previously, PFB also increased the cecum weight in all rats independent of diabetes, implying that the gut flora was altered by PFB, at least when added directly to the diet mix as powder [as in this study] or drink, but surprisingly NOT when supplemented in the drinking water as the only source of PFB.

**Figure 9:**
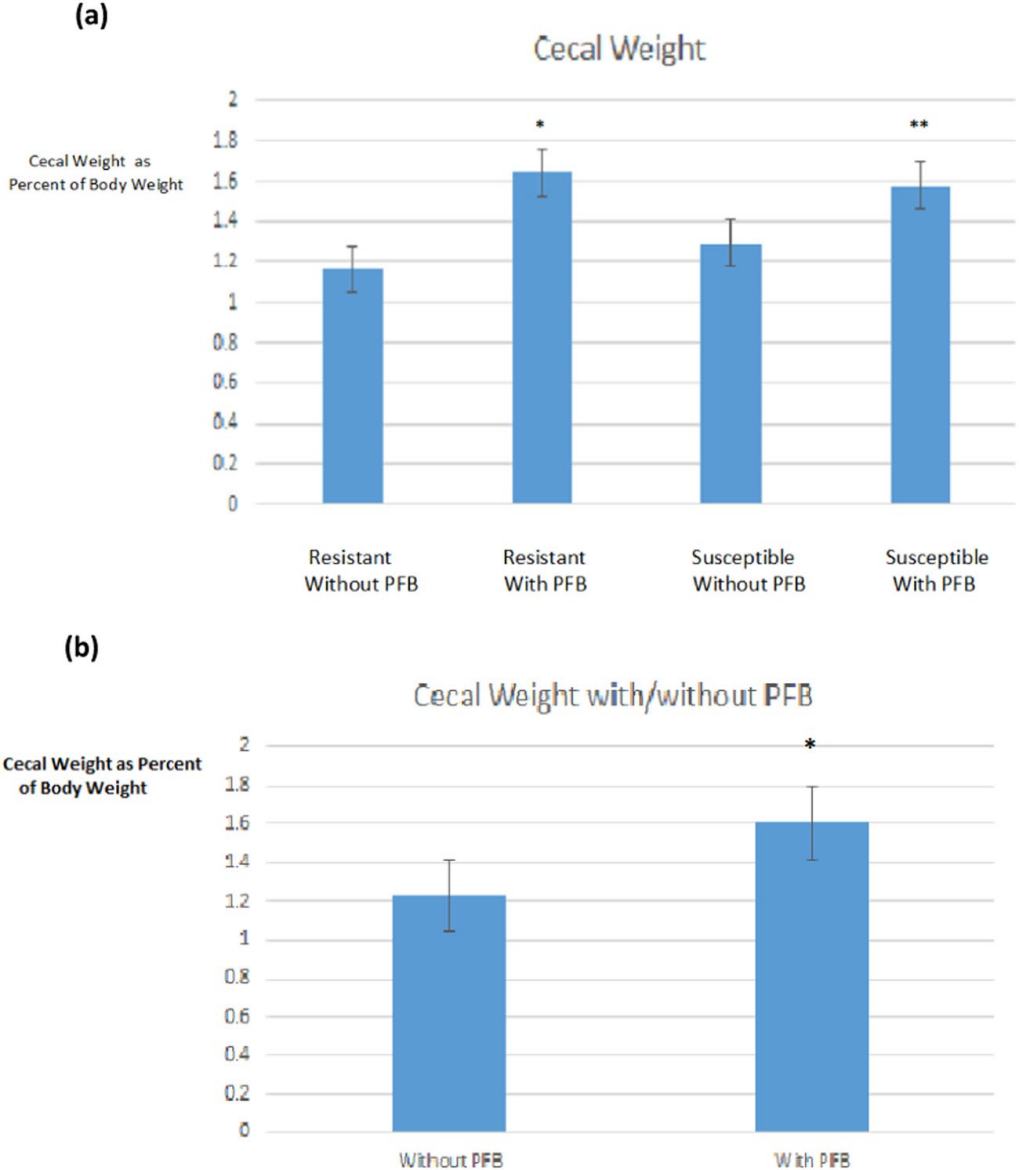
Cecal weights of NGR as percentage of total body weight. (**a**) DR = diabetes-resistant, DS = diabetes-susceptible, (−) PFB = not supplemented with PFB, (+) PFB = supplemented with PFB; (**b**) no PFB – not supplemented with PFB, With PFB – supplemented with PFB.

The mean cecum weight (as percentage of total body weight) of the diabetes-resistant rats not receiving PFB was 1.16% and of the diabetes-susceptible rats not receiving PFB was 1.29% whereas the mean cecum cecum weight of diabetes-resistant rats receiving PFB was 1.64% and of the diabetes-susceptible rats receiving PFB was 1.58% (Fig. 9A). These differences are statistically significant by two-way ANOVA. They also corroborate earlier gross observations by the Abeywardena Lab at CSIRO where at necropsy they noted that rats receiving PFB had grossly distended and discolored cecums. Figure 9A compares the diabetes-resistant and diabetes-susceptible NGR, which did or did not receive PFB (4 groups). Panel 9B compares all NGR which did not receive PFB pooled together with all NGR which did receive PFB pooled together.

We observed that the cecum size was about 33% greater for all rats which received PFB supplementation compared to those which did not receive PFB supplementation (Fig. 9B). This represents an increase in cecal weight of one-third of the basal level in the control NGR.

Cecum weight is primarily composed of its bacterial content. In essence, this suggests that increased proliferation of the microbiota (bacterial load) in diabetes-*susceptible* control rats [without   PFB] exacerbated the diabetic severity [associated with the greater cecum weight], while the supplementation of the diet with   PFB also induced greater cecal flora activity [as Cecum weight is associated with bacterial load], but here was beneficial in nature because the diabetic severity was reduced. In other words, more diabetes-inducing ‘bad flora’ prevailed with a hiCHO diet alone to induce diabetes, while enhanced-activity of gut flora with   PFB supplementation induced an anti-diabetic activity via a ‘good flora’ population. The Hayes Lab has performed some preliminary microbiome profiling with 16S rRNA gene sequencing which shows that there is a reversal in the Firmicutes/Bacteroides ratio between the diabetic versus non-diabetic NGR rats as well as distinct bacterial species which are different between the two groups (KC Hayes, personal communication).

## Discussion

Initially our studies were driven by epidemiologic data showing an association between diabetes and Alzheimer’s disease in humans so we focused on the hypothesis that greater diabetes-related neuropathology would exist in the brains of *diabetic-susceptible* NGR rats fed hiCHO diets compared with *diabetic-resistant* NGR rats fed the same diet, and that these distinctions would be enhanced by PFB as it protected against diabetes. Based on previous studies which have demonstrated the glucose-lowering effects of PFB in this model, which was coupled with modulation of lipid and insulin metabolic profiles in the Nile Grass rats, the questions raised were whether NGR rat brains would show similar signs of neuroinflammation and neurodegenerative changes characteristic of Alzheimer’s disease attributable to T2DM in humans and whether such neuropathology might be mitigated by a dose of PFB known to deter their T2DM.

However, the null hypothesis was not rejected because the brains from diabetic NGR rats revealed no neuro-pathologic characteristics compared with the non-diabetic NGR rats, looking at beta-amyloid, phosphorylated tau or alpha-synuclein by IHC. The low but detectable levels of these neuro-peptides were relatively the same for both diabetic and non-diabetic animals. However, surprisingly our panel of primary antibodies did identify a substantial difference in the IHC staining for TH, a key enzyme in catecholamine biosynthesis, when PFB was supplemented in the diet. . The blood glucose (RBG, FBG) and diabetic status had no effect on the expression of TH in these NGR rat brains, but only supplementation of the diet with 10% PFB in the hiCHO diet significantly increased tyrosine hydroxylase expression in the basal ganglia of these NGR rat brains, regardless of their diabetic metabolic state. The three areas of the NGR rat brain with the greatest increase in tyrosine hydroxylase were the striatum, caudate-putamen and the substantia nigra. Measurement of catecholamine levels in these areas of the NGR rat brain to assess the relationship between the elevated tyrosine hydroxylase levels and brain catecholamine levels await future studies now planned.

Several mechanisms may potentially explain the increase in brainstem tyrosine hydroxylase induced by PFB. Leow et al have previously shown that PFB specifically increased gene expression of the brain-derived neurotrophic factor (BDNF) as measured in the mouse transcriptome as well as BDNF-related neural networks^64^ and these changes seen with BDNF may be related to the changes in TH reported here. Protection against oxidative stress is known to restore tyrosine hydroxylase activity and PFB has been shown to be a potent antioxidant^51,65^ as well as potent negative-modulator of astrocyte-mediated neuroinflammation via reduction of TNF-α, RANTES and IP-10 secreted by the activated astrocytes^54^. In a transgenic yeast rescue assay, PFB has been shown to inhibit the aggregation and cytotoxicity of overexpressed α-synuclein (Angela Botes and Christian Ruckert, Sinskey Lab, personal communication).

Furthermore, it has previously been reported that brain-derived neurotrophic factor (BDNF) increases tyrosine hydroxylase expression in striatal neurons^66^. BDNF works coordinately with partner molecules to initiate tyrosine hydroxylase expression in striatal neurons. It has also been reported that BDNF activates gene transcription of tyrosine hydroxylase^67^. Activation of tyrosine hydroxylase (TH) gene transcription was induced by BDNF and its selective inhibition through Ca^2+^ signals evoked via the N-methyl-D-aspartate (NMDA) receptor. Our group has previously shown that PFB up-regulates several CNS genes involved in neuronal network maintenance and signaling through its upregulation of the BDNF network via murine transcriptomic studies^64^. This may also explain the findings of PFB increasing the levels of TH in the NGR rat brain in this study.

Regarding the gastrointestinal changes induced by PFB as evidenced by increased cecal weight, recent studies have shown the marked influence of the colonic   microbiota and their  metabolites on the brain and behavior. There is considerable evidence to suggest a role for the gut microbiota in increasing tyrosine hydroxylase activity in the NGR rat brain. Short chain fatty acids produced by the gut  microbiota have been shown to increase the expression of tyrosine hydroxylase^68^. PFB has been reported to increase short-chain fatty acid concentrations in caecal digesta of Sprague-Dawley rats (Conlon, “Consumption of an extract of oil palm fruit promotes large bowel health in Rats,”  submitted)).

The increased cecum weights in the NGR that received PFB may provide some important clues on the mechanism of action of PFB on the colonic microbiome. The increased cecum weights in NGR receiving PFB suggest the presence of enhanced microbiome activity and growth, as many studies have shown that cecum weight is directly related to microbiome activity and is correlated with an increased GI bacterial load.

Multiple published studies have examined the association between the gut microbiome and the development of Parkinson’s disease^69–76^ (which is marked by a deficiency of DA in the substantia nigra usually due to reduced numbers of dopamine-producing neurons). A sample of these studies include the following. The microbiome has been shown to have a favorable impact on PD when the microbiota profile is enhanced by the Prevotella family, as Prevotellaceae were significantly less abundant in the feces of PD patients in one cited study. A low abundance of bacteria from the *Prevotella* genus is linked to Western diets, and are increased by plant-based diets. Low *Prevotella* is associated with decreased mucin synthesis and therefore increased gut permeability [leaky gut syndrome] is seen in another cited study. It is also an associated risk factor for low-grade systemic inflammation, which is often a factor in patients with PD. We are planning experiments to look at whether: (1) the gut flora in NGR is in fact favorably altered by PFB; (2) what are the specific changes in the bacterial species induced by PFB; and (3) whether these changes in the colonic microbiome can be shown to be strongly associated with the increased TH levels in the brainstem.

Another study cited demonstrated that transplantation of fecal samples from patients with PD or healthy controls into healthy, germ-free mice showed that those mice receiving human microbiome samples from patients with PD then developed signs of PD. Transplanting samples from healthy persons into similar mice did not trigger the development of PD symptoms. One cited epidemiologic study showed that human data from 197 patients with PD and 130 healthy controls found that patients with PD had evidence of imbalanced gut microbiomes (dysbiosis), in which some bacterial species existed in larger numbers and some in smaller numbers compared to the healthy controls.

Previous studies in the Hayes Lab have revealed distinct alterations of the colonic microbiota in the diabetes-susceptible versus the diabetes-resistant NGR (unpublished data, KC Hayes, personal communication). Early preliminary microbiome profiling with 16S rRNA gene sequencing shows that there is a reversal in the Firmicutes/Bacteroides ratio between the diabetic versus non-diabetic NGR rats as well as distinct bacterial species which are different between the two groups. This preliminary data needs further study to elucidate the changes in the microbiome to determine which bacterial species account for the increased cecum weight. Collaborators at CSIRO in Australia have conducted similar studies in the Sprague-Dawley rats and found that PFB increases cecal weight in that strain as well (Conlon “Consumption of an extract of oil palm fruit promotes large bowel health in rats”,   submitted). We are currently planning research to look for any evidence of inflammation of the bowel which may be the result of the altered colonic microbiota.

At this point, we have only carried out these studies with the Nile Grass rat. It would also be interesting to see if PFB affects the brain expression of tyrosine hydroxylase in other rat strains  and even whether PFB similarly increases the brain expression of tyrosine hydroxylase in other mammals. We believe that most of the bioactive properties observed are elicited by various components of PFB working in synergy and this is likely to be true for tyrosine hydroxylase activity as well. Future studies will look at individual components of PFB in an attempt to isolate specific biologic properties to specific components. We are also planning on measuring the catecholamine levels in the brains of NGR rats which receive PFB which would normally be expected to be elevated in those regions showing increased TH activity.

## Main Conclusions

These studies have revealed that rapid induction of diabetes for 8 weeks with a high carbohydrate diet in male weanling Nile Grass rats is not associated with changes in the typical biomarkers of neurodegenerative processes as measured by beta-amyloid deposits, tangles of phosphorylated tau, or alpha-synuclein deposits. Although PFB supplementation deterred the incidence of T2DM substantially, it had no detectable impact on any of these peptide biomarkers as detected by immunohistochemical staining.

A novel finding is that diet supplementation with 10% PFB significantly increased the level of tyrosine hydroxylase in the basal ganglia of all Nile rat brains, independent of diabetic status. This increase in brain tyrosine hydroxylase is a novel and potentially significant finding unique to PFB . The increases were most pronounced in the striatum, caudate-putamen and the substantia nigra. Tyrosine hydroxylase is a key and rate-limiting enzyme in catecholamine biosynthesis in the brain and adrenal medulla. The next logical question is whether the PFB-induced increase in tyrosine hydroxylase is accompanied by a parallel increase in specific catecholamines, such as dopamine, norepinephrine and epinephrine.

The gastro-intestinal tract finding of increased cecum weight caused by the PFB requires further investigation. The recent literature on colonic microbiome suggests a potent effect on the brain and behavior, sometimes mediated by metabolites produced by the microbiota  . Early preliminary data from 16S rRNA gene sequencing suggest changes in the Firmicutes/Bacteroides ratio and differences in distinct bacterial species but these preliminary findings require further investigation.

## Materials and Methods

### Animal care, safety, protocols and euthanasia methods

All methods and procedures involving the use of the Nile Grass rats were approved by the Brandeis University Institutional Animal Care and Use Committee, Brandeis University, Bernstein Marcus, Room 121, MS 116, 415 South Street, Waltham, Massachusetts USA, and all methods and experimental protocols were conducted in accordance with all relevant university guidelines and regulations. Ethical and humane care principles were observed at all times in all the experiments.

### Nile grass rat subgroups and their respective diets

Thirty-nine male Nile Grass rats were separated and weaned at 3 weeks and fed a semi-purified high-carbohydrate diet (60:20:20, CHO:fat:Protein % energy) previously shown to induce MetS and T2DM in the NGR^32,35,36^. Approximately half were supplemented with 10% (w/w) PFB as spray-dried powder incorporated in the diet to give a final concentration of 4.7 mg GAE/g diet for 8 weeks duration.

Measuring daily food intake provided necessary details of energy intake that were expressed ultimately as metabolic characteristics related to clinical outcomes, including total calories consumed, body weight gain, RBG and FBG as well as OGTT at 4wk and 8wk, and organ pathology at necropsy after 8 weeks. Collectively the data better express differences in genetic permissiveness underlying the disease process.

### Immunohistochemical staining method

The NGR rats were selected for neuro-histologic studies of the brain based on their diabetes susceptibility. The rat brains were rapidly fixed by intra-arterial perfusion with 4% paraformaldehyde in 0.1 M phosphate buffer pH 7.4. The brains were removed from the cranial vault surgically. Coronal sections were made using a No. 11 surgical blade and the sections were placed in histology cassettes for paraffin embedding.

The sections were initially flooded with a blocking solution of 10% normal rabbit serum (NRS) with 0.1% Triton X-100 dissolved in 0.1 M PBS. Following the blocking solution, the sections were incubated with the mouse anti-tyrosine hydroxylase primary antibody working solution (Novus Biologicals, Cat No. NB300-109) at 4 °C for 16–24 hours. The working solution was prepared from the mouse anti-tyrosine hydroxylase primary antibody, diluted 1:25 with a solution of 5% NRS/0.1% Triton X-100 dissolved in 0.1 M PBS. Following the washing away of primary antibody with 3 excess volumes of 10% normal rabbit serum (NRS) with 0.1% Triton X-100 dissolved in 0.1 M PBS (diluent) for 10 minutes, the secondary antibody was added. The secondary antibody consisted of affinity-purified rabbit anti-mouse IgG peroxidase-conjugated antibody (Jackson ImmunoResearch Laboratories, West Grove, PA) at a dilution of 1:50 in 5% NRS mixed with 0.1% Triton X-100 in 0.1 M PBS. The sections were incubated with the secondary antibody with at 4 °C for 2 hours on the shaker. Following the washing away of unbound secondary antibody with 3 excess volumes of 10% normal rabbit serum (NRS) with 0.1% Triton X-100 dissolved in 0.1 M PBS (diluent) for 10 minutes, peroxidase substrate was then added to form insoluble dark brown product precipitate which can be visualized with optical light microscopy. The peroxidase substrate was provided by the DAB Substrate Kit (Vector, Burlingame, CA).

Sections were then rinsed 4 times in 0.1 M Tris-buffered saline (TBS), then mounted on subbed slides, dehydrated with ethyl alcohol, defatted with xylene, and then cover-slipped for light microscopic analysis. A subset of sections was counterstained with hematoxylin (Vector, Burlingame, CA). At least five coronal sections of mouse brain were processed for IHC. These five coronal sections included cerebrum, midbrain, brainstem, cerebellum and medulla. For the control sections, normal mouse serum (Sigma, St. Louis, MO) was substituted in place of the primary mouse anti-tyrosine hydroxylase antibody. IHC staining was carried out in the MIT Koch Institute Histology Core.

### Microscopy

Optical transmission light microscopy was performed on a Nikon Labophot-2 microscope (Nikon Inc., Japan) at an objective magnification of 4x.

### Image analysis

The microscopic images were quantitatively analyzed using the Nikon NIS-elements BR software (V 2.03, Nikon Instruments, Melville, NY).

### Statistical analysis

Statistical analysis of the results was performed using the Student’s t-test and two-way ANOVA with SPSS software.

## Data Availability

All relevant data are included within the manuscript but can also be made available online or through electronic mail for anyone requesting it.
