# Publisher Correction: Palm Fruit Bioactives augment expression of Tyrosine Hydroxylase in the Nile Grass Rat basal ganglia and alter the colonic microbiome

**DOI:** 10.1038/s41598-020-60010-9

**Published:** 2020-02-13

**Authors:** Robert P. Weinberg, Vera V. Koledova, Avinaash Subramaniam, Kirsten Schneider, Anastasia Artamonova, Ravigadevi Sambanthamurthi, K. C. Hayes, Anthony J. Sinskey, ChoKyun Rha

**Affiliations:** 1 Department of Biology, Massachusetts Institute of Technology, Cambridge, Massachusetts 02139 USA; 2 Biomaterials Science and Engineering Laboratory, Massachusetts Institute of Technology, Cambridge, Massachusetts 02139 USA; 3 Department of Biology, Brandeis University, Waltham, Massachusetts 02453 USA; 4 Advanced Biotechnology and Breeding Centre, Malaysian Palm Oil Board, 6, Persiaran Institusi, Bandar Baru Bangi, 43000 Kajang, Selangor Malaysia

Correction to: *Scientific Reports* 10.1038/s41598-019-54461-y, published online 09 December 2019

The Article contains an error in Table 3, entitled “Semi-quantitative comparisons of CNS proteins by IHC staining.”

In the “Tyrosine Hydroxylase” row under the “DM-resistant, with PFB” column, the value of 7+ was erroneously omitted.

